# Gender, nutritional disparities, and child survival in Nepal

**DOI:** 10.1186/s40795-022-00543-6

**Published:** 2022-05-23

**Authors:** Jasmine Fledderjohann, Melanie Channon

**Affiliations:** 1 Department of Sociology, Lancaster University, Bailrigg, Lancaster, UK; 2 Department of Social & Policy Sciences, University of Bath, Bath, UK

**Keywords:** Nepal, Child health, Gender, Breastfeeding, Child feeding, Son preference

## Abstract

**Background:**

This paper examines seemingly contradictory evidence from extant research that son preference is high, but male disadvantage in mortality is increasing in Nepal. To do so, we documented the timing, geographic patterning, and extent of gendered patterns in mortality and feeding practices for children under-five.

**Methods:**

We applied pooled multilevel regression models and survival analysis to five rounds of data from Nepal’s nationally representative Demographic and Health Surveys (1996–2016). We controlled for potential sociodemographic confounders, including child, maternal, household, and regional correlates, and disaggregated findings by birth order and sibling gender.

**Results:**

We found evidence of regional variation in mortality, with girls in wealthy urban areas faring the worst in terms of mortality rates. Girls’ comparative mortality advantage compared to boys in the neonatal period masks their mortality disadvantage in later periods. Mortality has fallen at a faster rate for boys than girls in most cases, leading to widening of gender inequalities. We also found evidence of female disadvantage in breastfeeding duration, which was linked to higher mortality risks, but no gender disparities in the consumption of other food items. Sibling gender and birth order also mattered for breastfeeding duration: Young girls with older sisters but with no brothers were most disadvantaged.

**Conclusion:**

While we did not find evidence of postnatal discrimination in access to solid and semi-solid foods, girls in Nepal face a disadvantage in breastfeeding duration. Girls with older sisters but no older brothers facing the greatest disadvantage, with risks being particularly concentrated for girls aged 1–4 years. This disadvantage is linked to an increased risk of mortality. To address this, community-based health programs could be expanded to continue targeted healthcare for children beyond 12 months of age, with particular focus on nutrition monitoring and health service provision for girls.

**Supplementary Information:**

The online version contains supplementary material available at 10.1186/s40795-022-00543-6.

## Background

Son preference has been documented in many low- and middle-income countries (LMICs). Consequences for girls include sex-selective abortions, discriminatory feeding practices, and mortality. Despite consistent evidence of strong son preference in Nepal [1–4], however, Nepali boys face a survival disadvantage compared to girls [5]. While prenatal discrimination has been well-documented in Nepal [3, 6, 7], patterns of postnatal discrimination are under-researched. In this paper, we examine gendered nutrition and mortality outcomes for children aged under-five.

### Nutrition and survival

Nutrition plays a central role in shaping mortality risks in childhood. Children experiencing severe micronutrient deficiencies and/or severe undernutrition (i.e. stunting, wasting, underweight) face significant risks of infectious illnesses, organ failure, and mortality [8–10]. UNICEF [11] estimates undernutrition contributes to more than one-third of under-five mortality globally. To combat early childhood undernutrition, exposure to waterborne pathogens, and diarrheal disease, WHO recommend introduction of breastfeeding within 1 h of birth, exclusive breastfeeding until 6 months, introduction of complementary foods at 6 months, and continued breastfeeding for at least 24 months [12]. Once complementary feeding begins, children should eat a diverse diet to prevent micronutrient deficiencies.

Parents may depart from best practices for many reasons, including limited or incorrect information about best practices; insufficient access to a reliable, nutritious supply of food; and/or uneven distribution of food due to personal preferences [13–15]. These possibilities are not mutually exclusive—parents may, for example, struggle to access nutritious food, and also not purchase available food because it is undesirable. In some cases, parents prefer packaged, processed foods from markets over fresh local food because packaged foods are perceived as healthier, reflecting both knowledge and preferences [16]. Distal factors impacting undernutrition include household poverty, parental education, geospatial distribution of infectious diseases, food prices, and state policies [13, 15, 17–19].

Child nutrition is also shaped by intra-household dynamics. One recent study [20] from Dhanusha and Mahottari districts in Nepal considered whether maternal dietary intake and nutritional status and sex differences in growth in the first 20 months of life accounted for the observation that boys grow faster but are more susceptible to undernutrition compared to girls. They found that maternal anthropometry and dietary intake did not differ according to child sex, neither during pregnancy nor in the postpartum period, but that comparable maternal restricted food intakes during pregnancy may negatively impact fetal growth for boys more than for girls. Some literature, mainly from India, suggests girls may be given less food/less preferred foods where competition with brothers for nutritional resources is high [21–23]. However, evidence is mixed, with other research finding limited evidence of preferential treatment of boys [24–27]. Further research is needed to understand when/how/for what nutritional resources sibling competition intersects with gender to produce nutritional inequalities—particularly in Nepal, where literature on this is scarce.

### Son preference

Son preference has wide-ranging negative consequences for girls, including sex-selective abortion [3, 7, 28, 29]; poorer investments in prenatal treatment when fetal sex is known [7, 30]; restricted access to healthcare [31]; shorter breastfeeding duration [22–24, 32]; and lower immunization rates [32]. Where sons are preferred, couples who have daughters may try again for a son even where they have met or exceeded their ideal family size. Son preference thus results in two aggregate demographic trends [33]: a sibling effect, in which girls tend to be born into larger families, and a birth-order effect, in which girls tend to be earlier in the birth-order than boys. Importantly, however, son preference is not universal: As Sapkota et al. [34] show, son preference is greater among women who are less educated, poorer, and/or who live in rural areas. They also document variation in son preference by ethnicity, with women from the Terai Brahman, Terai Dalit, Muslim, and other ethnic minority groups expressing strong son preference compared to women from other ethnicities.

Evidence of son preference in Nepal has centered on stopping behavior, contraceptive use, and sex-selective abortion [3, 4]. There has been less work on postnatal discrimination. In Nepal, though the reported desired sex ratio at birth has decreased since 1996 [2], demographic manifestations of son preference have increased. Most notably, there is evidence of growing sex-selective abortion, especially in the Kathmandu Valley and amongst wealthier, more educated women [3]. While Leone et al. [35] found the effects of son preference on fertility and contraceptive use in 1996 were moderate, more recent evidence shows the effects have increased over time [2, 4].

### Child health in Nepal

Nepal performed very well at meeting Millennium Development Goal 4, which called for a two-thirds reduction in mortality between 1990 and 2015 [36]. Nationally representative data from 2006 to 2016 show that, among Nepali children under the age of five, diarrhea and acute respiratory infections have declined (by 4% and 3% respectively), and treatment-seeking practices have improved over the period [37]. Despite these encouraging trends, sociodemographic and regional disparities in child health and survival have persisted, with children in poor households being particularly disadvantaged. Nonetheless, the under-five mortality rate (U5MR) fell by 72% since 1990, from 142 deaths per 1000 live births in 1990 down to 40 in 2013 [38]. Although this represents remarkable progress, Nepal still ranks 61st globally for highest U5MR, despite high childhood immunization rates and falling rates of low-birthweight (LBW) infants. One explanation may be the very high prevalence of childhood undernutrition. The most recent figures from the Nepal Demographic and Health Surveys (NDHS) in 2016 show that 36% of children under the age of five were stunted, and 27% were underweight [39]. These measures reflect long-term undernutrition, including through non-adherence to WHO recommended feeding practices. Some small-scale studies from Dolakha and Kavre districts and from Dukuchhap village in Lalitpur district focusing on early [40] and mid-childhood [41] suggest girls are more likely to experience sustained undernutrition than boys.

Sibling characteristics, birth spacing, and family sociodemographics can influence children’s health and nutrition. Using data from a randomized control trial of newborn infections in rural Southern Nepal, Rosenstock et al. [42] found that although there was no gender gap in neonatal mortality overall, within the neonatal period (first 28 days of life) boys faced a greater risk earlier in the period, while girls faced a greater risk of mortality later in this period. For boys, greater risk was explained by biological factors. For girls, sociodemographic factors drove elevated risk—particularly sibling gender and birth order, with girls with no brothers facing the greatest risk of mortality. Girls with no older brother are likely to experience a shorter succeeding birth interval as a result of son preference [43]. The birth intervals before and after the birth of a middle-child are negatively associated with child survival, with much of the association operating through early weaning [44].

Maternal age, education, and economic status all have a positive relationship with breastfeeding duration [45], and maternal socioeconomic status and ethnicity impact initiation of breastfeeding within one hour of birth [46, 47]. Mothers in Nepal’s mountains are more likely to initiate breastfeeding and complementary feeding following recommended practices, and mothers in urban areas are more likely to use bottle-feeding rather than breastfeeding [48].

### Study contributions

Although son preference is not the only driver of gendered outcomes, it is well-documented as a critical factor impacting child health in South Asia [22–24, 32]. There has, however, been a relative dearth of studies focused specifically on Nepal. Here, we examine gendered nutritional and mortality outcomes, asking:

1. What are the aggregate patterns of gender differences in mortality across time and place in Nepal?
2. Are girls disadvantaged in infant and early childhood feeding practices?
3. Where disadvantage exists, are feeding practices associated with mortality risks?

We question the notion that son preference has a uniform effect on female nutrition and survival, and consider how son preference interacts with birth order and sibling gender to shape health outcomes.

## Methods

### Data

We use 5 rounds of the NDHS [49], a publicly available, nationally representative cross-sectional survey conducted every 5 years since 1996. Surveys include women’s reports on maternal and household sociodemographics, reproductive histories, and child health [50]. Detailed data about breastfeeding and food consumption were collected only for children born within 36 months of the survey in 1996, and within 59 months of the survey in subsequent years. We therefore restricted the sample to children aged < 37 months in 1996, and < 60 months thereafter. Data from the 2011 Nepal Census were used to construct a map of female U5MR as a percentage of male U5MR across Districts of Nepal.

### Availability of data and materials

The datasets analysed for this study are available in the Demographic and Health Surveys repository, (https://dhsprogram.com/data/available-datasets.cfm). Data were publicly available and fully anonymized, and so ethical approval was not required.

### Dependent variables

**Fig. 1:**
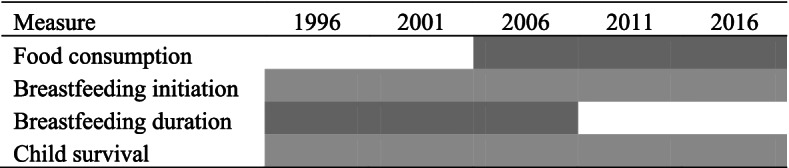
Availability of measures by survey round, NDHS 1996–2016

Because NDHS has undergone revisions over time, some measures are not available in all years. Figure 1 shows availability by survey round.

#### Food consumption

For living children, mothers reported whether children consumed (1 = yes) 16 different categories of food items within the last 24 hours, including roots and tubers, green leafy vegetables, meat and poultry, vegetarian proteins (e.g. legumes), dairy and grain products, and fats. Interviewers read out: “Now I would like to ask you about liquids or foods that (NAME) had yesterday during the day or at night. I am interested in whether your child had the item I mention even if it was combined with other foods. Did (NAME) drink or eat:” The interviewers then read out each item in the list and recorded whether mothers indicated yes, no, or don’t know. Items on the list included, for example “Any meat, such as pork, buff, lamb, goat, chicken, or duck,” “Any dark green, leafy vegetables like spinach,” “Pumpkin, carrots, squash, or sweet potatoes that are yellow or orange inside,” etc. Following WHO guidance for infant and young child feeding [12], we also used these variables to calculate dietary diversity scores for children over 6 months of age (before which exclusive breastfeeding is recommended) based on whether they consumed items in each of seven broad categories within the last 24 hours: grains, roots, and tubers; dairy; legumes and nuts; meat, fish, poultry, and animal organ meat; vitamin A-rich fruits and vegetables; other fruits and vegetables; and eggs. We coded children as having a diverse diet if they consumed foods from at least four of these groups.

#### Breastfeeding

For living children, mothers provided retrospective reports of whether breastfeeding was initiated within 1 hour of birth (1 = yes). NDHS do not include a question about duration of exclusive breastfeeding; we were unable to examine exclusivity. We examine breastfeeding duration based on mothers’ replies to whether they ever breastfed each child; mothers responding ‘yes’ were then asked, “Are you still breastfeeding (NAME)?” Where women answered ‘yes’, breastfeeding duration was coded as equal to the child’s current age in months. Mothers of weaned children were asked “For how many months did you breastfeed (NAME)?” This was asked for all children < 36 months in 1996, and all children < 60 months in 2001 and 2006. Unfortunately, from 2011 a survey revision means it is only possible to calculate duration for currently breastfeeding children.

#### Child survival

We utilize a dichotomous indicator of whether the child was living at the survey date (yes = 1). For children who died, we calculated survival duration by subtracting date of birth from date of death, reported retrospectively by mothers.

### Independent variables

Our key independent variable is child’s gender (female = 1). We also consider sibling gender and birth order because these factors may shape child health outcomes [23, 24, 32, 33]. We measure birth order and sibling gender in 5 categories: [1] firstborn [2]; second or thirdborn with no older brothers (e.g. a secondborn girl following a firstborn girl) [3]; second or thirdborn with an older brother (e.g. a secondborn girl following a firstborn boy) [4]; fourth-or-higher birth order with older brother(s) [5]; fourth-or-higher birth order with no older brothers. To account for sociodemographic factors which may correlate with feeding practices and son preference, we include maternal age and education (no education; completed primary or less; attended or completed secondary; and attended or completed higher education), urban versus rural residence, household poverty (household wealth in the bottom two quintiles on the NDHS wealth index), household size, religion (Hindu = 1), ethnicity (Brahaman/Chhetri; Tarai/Madhesi other castes; Dalits; Newar/Janajati; and Muslim/Other), and ecological region (Hills, Terai, or Mountain). Hazard models for breastfeeding also include a control for whether the child was alive at the time of the survey. Our models focusing on children’s food consumption also include child age (in months). Additionally, given evidence that seasonality is associated with child mortality [51] and dietary intakes [20] in some areas of Nepal (studies from rural Sarlahi and rural areas of Dhanusha and Mahottari districts respectively), we also included a control for interview month in our models of child survival and food consumption.

### Analytic strategy

We tested (1) baseline models with sociodemographic controls, (2) models with the five-category birth order/sibling gender predictor (child birth order excluded), and (3) models including an interaction between child gender and the five-category birth order/sibling gender variable. Our pooled sample across 5 rounds of data included *n* = 25,648 children born to *n* = 19,135 mothers within *n* = 17,560 households. Missing data were handled using listwise deletion.

We applied pooled multilevel logit regression models to analyse breastfeeding initiation and food consumption accounting for clustering of children within households. Due to variation in sampling frame and survey questions between years, we ran separate models for breastfeeding duration in 1996 and 2001. However, as the questionnaire and sample design matched in 2001 and 2006, we pooled models in these years. Pooling data where possible allowed us to increase sample size and reduce standard errors, improving the precision of our estimates [52]. In pooled models, we included a control for year to capture potential changes in health and feeding practices over time. Gender differences in initiation and individual food item consumption were estimated using separate multilevel logit regressions for each outcome.

We also applied discrete-time hazard models to estimate the hazard of cessation of breastfeeding and child survival. Discrete-time models were selected because these outcomes are measured as discrete monthly data. Hazard models handle censoring well, so the restriction of the sample to children under 36 months in the 1996 survey is unproblematic. There were not enough observations in some months to fit the models using 1-month intervals, so we created 3-month time periods. Truncation occurred after 16 time points for models of breastfeeding duration, and 19 points for child survival. For interpretability, we illustrated the significant interaction between gender and five-category birth order/sibling gender using Kaplan Meier curves. Models were fitted using Stata version 13.1.

## Results

### Descriptive statistics

Breastfeeding practices appear to be improving over time (Table 1). In 1996, a little over one-third (37.4%) of the sample was put to breast within 1 h of birth. This figure increased steadily, rising to nearly half (49.1%) in 2001, 57.9% in 2006, nearly three quarters (69.6%) in 2011 and 2016 (73.5%). Breastfeeding duration also rose, from a mean duration of 15.2 months in 1996 up to 26.0 months in 2011. While figures for 2011 and 2016 should be interpreted with caution as they are only for currently breastfeeding children, these patterns overall point to improvements in adherence to WHO recommendations. A similar pattern of improvement held for child survival: while 92.8% of children were currently living in the 1996 survey, this figure rose over the period, up to 96.6% in 2016.

**Table 1:** Descriptive statistics for analytic samples, NDHS 1996–2016

	1996			2001			2006			2011			2016		
Variable	n	Mean or %	St. Dev.	n	Mean or %	St. Dev.	n	Mean or %	St. Dev.	n	Mean or %	St. Dev.	n	Mean or %	St. Dev.
Breastfed within 1 Hour	3615	37.4%	0.48	4315	49.1%	0.50	4976	57.9%	0.49	4990	69.6%	0.46	4667	73.5%	0.44
Breastfeeding Duration	4038	15.2	9.43	6247	20.8	12.71	5385	21.4	12.75	3688	26.0	16.60	2736	20.5	13.61
Child is Alive	4129	92.8%	0.26	6371	92.7%	0.26	5465	94.4%	0.23	5004	95.4%	0.21	4674	96.6%	0.18
Child is Female	4129	48.5%	0.50	6371	49.9%	0.50	5465	49.6%	0.50	5004	47.7%	0.50	4674	47.1%	0.50
Child’s Age in Months	4129	16.4	10.61	6371	28.2	17.91	5465	29.0	17.78	5004	28.9	17.85	4674	29.5	17.67
Birth Order & Sibling Gender															
Firstborn	4129	21.3%	0.41	6371	22.1%	0.41	5465	28.4%	0.45	5004	31.6%	0.46	4674	38.1%	0.49
2nd/3rd Born No Brothers	4129	8.1%	0.27	6371	9.1%	0.29	5465	11.3%	0.32	5004	12.1%	0.33	4674	13.1%	0.34
2nd/3rd Born Has Brother(s)	4129	30.0%	0.46	6371	30.4%	0.46	5465	31.1%	0.46	5004	32.5%	0.47	4674	31.1%	0.46
4+ Born No Brothers	4129	1.8%	0.13	6371	2.1%	0.14	5465	2.2%	0.15	5004	2.0%	0.14	4674	2.1%	0.14
4+ Born Has Brother(s)	4129	38.8%	0.49	6371	36.3%	0.48	5465	27.0%	0.44	5004	21.8%	0.41	4674	15.6%	0.36
Maternal age	4129	27.0	6.44	6371	28.0	6.40	5465	27.1	6.15	5004	27.1	5.96	4674	26.5	5.49
Maternal Education															
None	4129	80.8%	0.39	6371	75.2%	0.43	5465	62.0%	0.49	5004	47.8%	0.50	4674	33.9%	0.47
Primary	4129	10.6%	0.31	6371	13.5%	0.34	5465	17.5%	0.38	5004	19.4%	0.40	4674	19.9%	0.40
Secondary or Higher	4129	8.6%	0.28	6371	11.3%	0.32	5465	20.4%	0.40	5004	32.7%	0.47	4674	46.3%	0.50
Urban	4129	8.5%	0.28	6371	8.3%	0.28	5465	22.3%	0.42	5004	20.3%	0.40	4674	56.8%	0.50
Poor Household	4129	46.4%	0.50	6371	48.0%	0.50	5465	50.9%	0.50	5004	52.8%	0.50	4674	48.7%	0.50
Household Size	4129	7.4	3.81	6371	7.1	3.41	5465	6.6	3.21	5004	6.0	2.59	4674	6.1	2.90
Hindu	4129	86.0%	0.35	6371	83.8%	0.37	5465	87.0%	0.34	5004	85.7%	0.35	4674	87.1%	0.34
Caste/Ethnicity															
Brahaman/Chhetri	4129	31.7%	0.47	6371	32.2%	0.47	5465	33.6%	0.47	5004	38.0%	0.49	4674	33.6%	0.47
Tarai/Madhesi Other Castes	4129	9.2%	0.29	6371	11.8%	0.32	5465	11.7%	0.32	5004	7.2%	0.26	4674	14.8%	0.35
Dalits	4129	15.9%	0.37	6371	15.7%	0.36	5465	16.7%	0.37	5004	18.4%	0.39	4674	15.0%	0.36
Newar/Janajati	4129	38.2%	0.49	6371	35.2%	0.48	5465	33.3%	0.47	5004	32.2%	0.47	4674	30.4%	0.46
Muslim/Other Caste	4129	5.0%	0.22	6371	5.2%	0.22	5465	4.7%	0.21	5004	4.2%	0.20	4674	6.2%	0.24
Region															
Mountain	4129	14.5%	0.35	6371	15.8%	0.36	5465	15.4%	0.36	5004	19.7%	0.40	4674	9.2%	0.29
Hill	4129	41.8%	0.49	6371	37.6%	0.48	5465	39.0%	0.49	5004	40.5%	0.49	4674	42.3%	0.49
Terai	4129	43.8%	0.50	6371	46.6%	0.50	5465	45.6%	0.50	5004	39.9%	0.49	4674	48.5%	0.50
Interview Month	4129	8.4	4.39	6371	6.8	4.81	5465	5.3	4.07	5004	7.1	4.91	4674	6.1	2.04

Note: Sample sizes for breastfeeding variables are smaller than those for sociodemographics because these questions were not asked for all children due to legitimate skips (e.g., valid missing for children who had never been breastfed)

Around 50% were female in each round. Mean child age was around 29 months in most years.^1^ The percent of children with no brothers was substantially lower than those with brothers. Mean maternal age ranged from 26.5 to 28.0 years across surveys. Education increased substantially: 80.8% of mothers had no formal education in 1996, compared to 33.9% in 2016. Secondary or higher education climbed from 8.6% in 1996 to 46.3% in 2016. There was a noticeable jump in the percent urban across rounds, with 8.5% of the sample living in urban areas in 1996 and 2001 compared to around 20% in 2006 and 2011, with another large jump up to 56.8% in 2016; this latter jump corresponds to governmental reclassification of administrative boundaries rather than large-scale migration. Household size declined somewhat over time, from a mean of 7.4 household members in 1996 down to 6.1 in 2016. Across 1996 through 2011, a majority (~ 85%) of the sample was Hindu. Caste/ethnicity fluctuated slightly across rounds, but in general around one-third of respondents were Brahaman/Chhetri and another roughly one-third were Newar/Janajati. The next largest group were Dalits, around 15% in most rounds, followed by Tarai/Madhessi Other Castes. The smallest caste/ethnicity group, around 5% across rounds, were Muslim/Other respondents. A minority (from 14.5 to 19.7%) lived in the mountain region, while around 40% each lived in the hill and terai regions. In 2016 the lowest proportion of respondents across rounds lived in the mountain region (9.2%), and the highest lived in the terai region (48.5%). There was some variation in when interviews took place across surveys, but in general on average surveys occurred in spring and summer months.

### Mortality

**Fig. 2:**
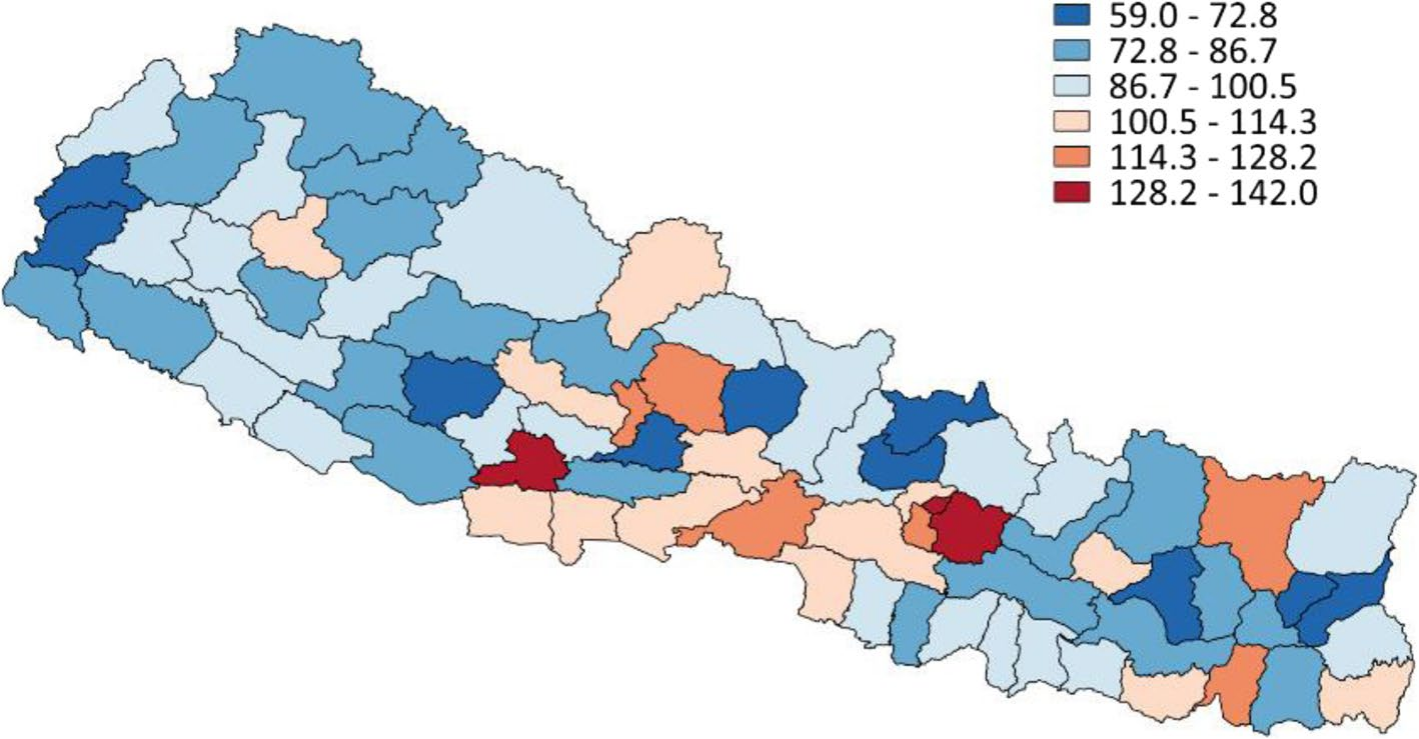
Female U5MR as a percentage of male U5MR by district, Nepal 2011 Census

Figure 2 shows female U5MR as a percentage of male U5MR by district (*n* = 75) according to 2011 population Census data. The 24 red districts have a higher female U5MR—that is, where female U5MR exceeds male U5MR and the sex ratio therefore exceeds 100. As Costa et al. [53] note, boys experience greater biological frailty in early childhood, and higher female mortality ratios run counter to girls’ biological survival advantage in early life, indicating gender-based inequalities. From a descriptive overview of Fig. 2, districts with higher female mortality tended to be richer and more urban areas, with lower mortality overall. Table 2 provides results from the NDHS by gender and year for the neonatal (first 28 days), post-neonatal (1–12 months), infant (first 12 months), child (1–4 years), and under-five mortality rates. The female child mortality rate was generally higher than the male child mortality rate in all surveys. Likewise, the female post-neonatal mortality rate was higher than the male post-neonatal mortality rate across time periods. However, this later female disadvantage was masked in the U5MR for 2011 and 2006 by the mortality disadvantage faced by boys in the neonatal period. Girls also had a lower mortality rate than boys in the neonatal period but a higher rate in subsequent periods in 2016; however, this period-specific pattern did not mask girls’ overall mortality disadvantage. In fact, in 2016 girls had a higher U5MR than boys for the first time since 2001.

**Table 2:** Mortality rates over time by gender, NDHS, 1996–2016

	Neonatal	Post-Neonatal	Infant	Child	Under-Five
2016					
Male	24	7	31	5	36
Female	17	17	34	7	41
2011					
Male	37	17	54	9	63
Female	33	19	52	10	62
2006					
Male	39	21	60	21	80
Female	37	24	61	18	78
2001					
Male	52	27	79	28	105
Female	43	32	75	40	112
1996					
Male	66	36	102	46	143
Female	50	33	84	57	136

Across outcomes, mortality rates improved, and the gender gap narrowed between 1996 and 2016. Specifically, U5MR rates for boys dropped by 27% between 1996 and 2001, 24% from 2001 to 2006, and 21% from 2006 to 2011. Corresponding figures for girls were declines of 18%, 30%, and 21% respectively—rates declined faster for boys than girls in earlier periods, but declines in U5MR were at parity by 2011. While the 2016 data show a substantial drop in the U5MR for both boys (43% decrease from 2011) and girls (34%), mortality rates for girls again declined at a slower rate—a return to the pattern in previous periods. Girls were particularly disadvantaged in the rate of mortality decline in the post-neonatal period, when boys’ mortality dropped by 59%, compared to only 11% for girls.

### Food consumption

We found no significant gender differences in food consumption for any of the 16 reported food items in any round, nor for dietary diversity. Results are not shown for brevity, but were tested in separate logit regressions for each food item.

### Breastfeeding

We found a marginally significant association between child gender and breastfeeding initiation (Appendix Table S1), with girls having slightly lower odds than boys of being breastfed within 1 h of birth (OR = 0.82; *p* < 0.01). This association reduced slightly (OR = 0.82; *p* < 0.05) once birth order and sibling gender were taken into account. All children of higher birth orders had higher odds of being put to breast within an hour of birth as compared to firstborns; sibling gender did not make a substantial difference. We did not find evidence of a significant interaction between child gender and sibling gender and birth order.

However, child gender was a strong, significant predictor of breastfeeding duration. Table 3 provides results for discrete-time hazard models predicting breastfeeding duration. Model 1 shows, accounting for all baseline controls, girls faced a higher hazard of breastfeeding cessation compared to boys (HR = 1.16; *p* < 0.001), i.e. had shorter breastfeeding duration. Higher order births were associated with a longer duration of breastfeeding. The association between gender and breastfeeding cessation was increased (HR = 1.22; *p* < 0.001) by the inclusion of the five-category birth order/sibling gender variable (Model 2). All groups had a lower hazard of breastfeeding cessation than firstborns, and girls with older brothers had a lower hazard of breastfeeding cessation than girls with only sisters. Girls of birth order four or higher who had older brothers faced the lowest risk.

**Table 3:** Pooled discrete-time hazard models of breastfeeding cessation, all living children, NDHS 1996–2006

	Model 1		Model 2		Model 3	
	OR	95% CI	OR	95% CI	OR	95% CI
Child is Female	1.16^***^	[1.08,1.24]	1.22^***^	[1.14,1.31]	1.09	[0.96,1.24]
Child’s Birth Order						
1st Born (ref)						
2nd/3rd Born	0.72^***^	[0.65,0.79]				
4th + Born	0.59^***^	[0.53,0.66]				
Birth Order & Sibling Gender						
1st Born (ref)						
2nd/3rd Born, No Brothers			0.84^**^	[0.74,0.95]	0.69^***^	[0.60,0.80]
2nd/3rd Born, 1+ Brother(s)			0.63^***^	[0.58,0.69]	0.61^***^	[0.54,0.71]
4th + Born, No Brothers			0.65^**^	[0.51,0.84]	0.57^***^	[0.42,0.76]
4th + Born, 1+ Brother(s)			0.49^***^	[0.43,0.56]	0.48^***^	[0.41,0.56]
Interactions						
Female*2nd/3rd Born, No Brothers					2.13^***^	[1.65,2.76]
Female*2nd/3rd Born, 1+ Brother(s)					1.07	[0.90,1.28]
Female*4th + Born, No Brothers					1.80^*^	[1.02,3.18]
Female*4th + Born, 1+ Brother(s)					1.06	[0.89,1.27]

Notes: Models control for maternal age and education, urban residence, region, household poverty, household size, year, ethnicity, and religion; constant estimated but not reported; * *p* < 0.05, ** *p* < 0.01, *** *p* < 0.001

Model 3 showed a significant interaction between child gender and birth order/sibling gender. The main effect of being female (HR = 1.09; *p* > 0.05) was non-significant; however, this is because of the very strong and significant interaction between child gender and our five-category measure. Female disadvantage in breastfeeding for higher order births was stark. Older brothers had a significant effect on breastfeeding duration, but only for girls. A second- or thirdborn girl with no brothers had increased odds of breastfeeding cessation compared to a firstborn male. Second- or thirdborn girls with older brothers were actually breastfed longer than firstborn males, but not as long as second- or thirdborn males. This is consistent with the idea that at low parity, women plan to have another child regardless of child sex, and so stop breastfeeding early. At higher parity, progression decisions are based on number and gender composition of existing children.

**Fig. 3:**
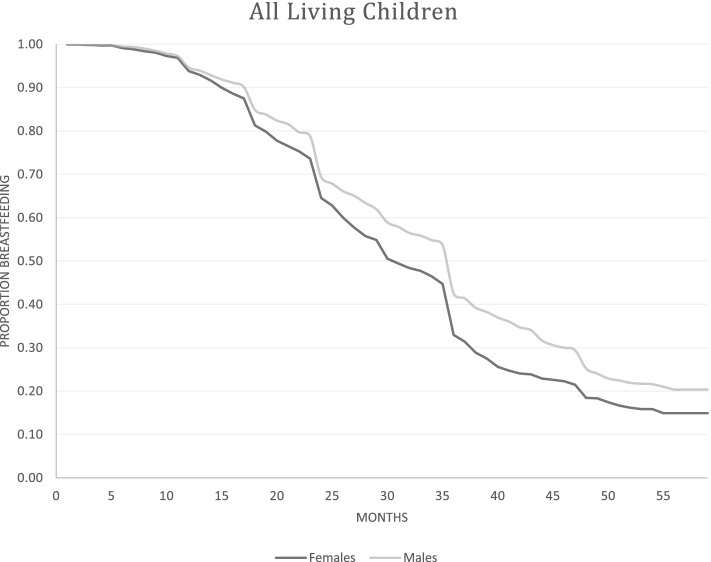
Kaplan Meier Curves for breastfeeding duration for all living children, NDHS 1996–2006

**Fig. 4:**
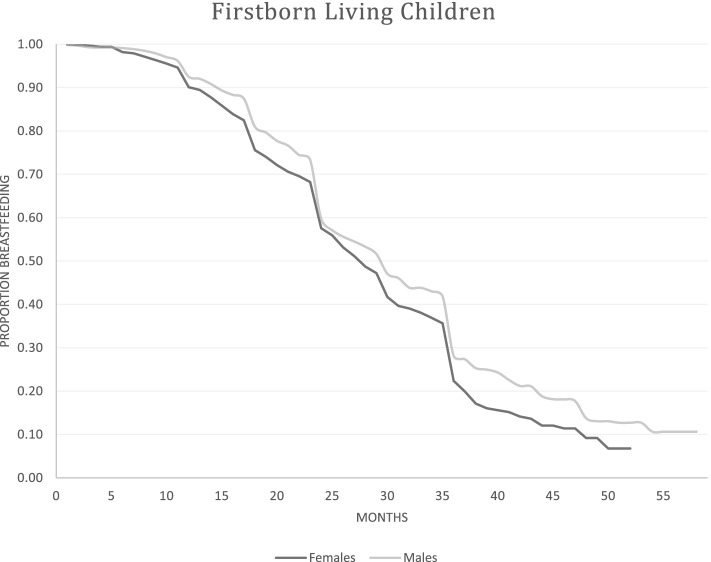
Kaplan Meier Curves for breastfeeding duration for firstborn living children, NDHS 1996–2006

**Fig. 5:**
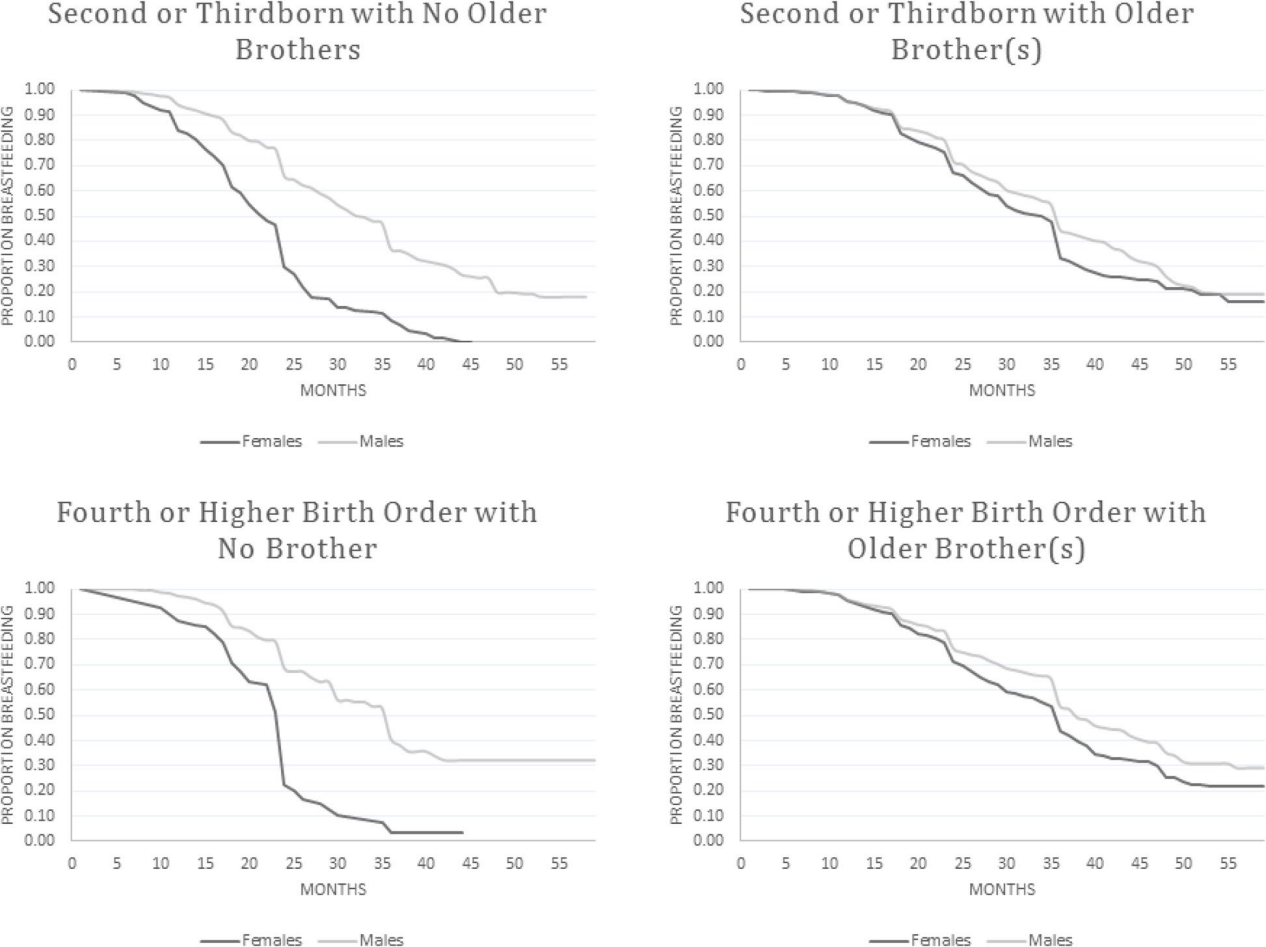
Kaplan Meier Curves for breastfeeding duration for second and higher birth order living children, NDHS 1996–2006

To ease interpretation of interaction effects from these models, we produced Kaplan Meier curves, presented in Figs. 3-5. They show the proportion still breastfeeding on the Y-axis, and time in months along the X-axis. The trend for boys is given by the light grey line, and for girls by the darker line. Figure 3 shows all living children in the pooled sample. A slight gender gap emerged around 12 months, growing with child age. There were three large drops in breastfeeding, around 12, 24, and 36 months, consistent with normative age-based weaning practices. The trend for firstborns closely resembled the overall trend for all children (Fig. 4). There was again an observable female disadvantage in breastfeeding duration. It emerged earlier than in the overall trend, but was still small.

A markedly different pattern emerged at higher birth orders. The top left panel of Fig. 5 shows trends for second- and thirdborn children with no older brothers. Female disadvantage in breastfeeding duration appeared around 6 months and grew rapidly, resulting in a very large gap by 24 months. No girls were still breastfeeding past 45 months, while nearly 20% of boys were still breastfeeding at the final observation point. The gender gap between second- and thirdborn boys and girls with 1+ older brothers was small and emerged later (around 12 months), shown in the top right panel of Fig. 5. Patterns for fourth-or-higher order births without (bottom left) and with (bottom right) 1+ older brothers were similar to those for second- and thirdborn children.

### Mortality

To assess the link between female disadvantage in breastfeeding and child survival, Table 4 provides results for the pooled discrete-time hazard models predicting the hazard of mortality. Model 1 shows that, controlling for sociodemographic variables, every month of additional breastfeeding conferred an approximately 4% lower hazard of death (HR = 0.96; *p* < 0.001). This relationship held across Models 2 and 3, with controls for sibling gender and birth order variables and the interactions with gender respectively. Thus, the key gendered nutrition outcome documented in our study was strongly, significantly linked to child survival. However, this did not counterbalance the survival advantage for girls overall—girls had a lower hazard of mortality compared to boys (Model 1: HR = 0.94; *p* < 0.001; Model 2: HR = 0.91; p < 0.001; Model 3: HR = 0.93; *p* < 0.05).

**Table 4:** Pooled discrete-time hazard models of child survival, all children, NDHS 1996–2006

	Model 1		Model 2		Model 3	
	HR	95% CI	HR	95% CI	HR	95% CI
Breastfeeding duration (months)	0.96^***^	[0.96,0.97]	0.96^***^	[0.96,0.97]	0.96^***^	[0.96,0.97]
Child is Female	0.94^***^	[0.90,0.97]	0.91^***^	[0.88,0.95]	0.93^*^	[0.87,0.99]
Child’s Birth Order	0.67^***^	[0.63,0.70]				
1st Born (ref)	0.70^***^	[0.66,0.74]				
2nd/3rd Born						
4th + Born						
Birth Order & Sibling Gender						
1st Born (ref)						
2nd/3rd Born, No Brothers			0.59^***^	[0.55,0.63]	0.63^***^	[0.58,0.68]
2nd/3rd Born, 1+ Brother(s)			0.71^***^	[0.67,0.75]	0.70^***^	[0.65,0.75]
4th + Born, No Brothers			0.62^***^	[0.54,0.72]	0.66^***^	[0.57,0.77]
4th + Born, 1+ Brother(s)			0.77^***^	[0.72,0.83]	0.76^***^	[0.70,0.83]
Interactions						
Female*2nd/3rd Born, No Brothers					0.62^***^	[0.51,0.76]
Female*2nd/3rd Born, 1+ Brother(s)					1.02	[0.93,1.12]
Female*4th + Born, No Brothers					0.63^*^	[0.41,0.97]
emale*4th + Born, 1+ Brother(s)					1.02	[0.93,1.12]

Notes: Models control for maternal age and education, child age, urban residence, region, household poverty, household size, survey year, interview month, ethnicity, and religion; * *p* < 0.05, ** *p* < 0.01, *** *p* < 0.001

### Robustness checks

Having an older brother and having a *living* older brother may differ in terms of discrimination and feeding practices. As a robustness check, we created an additional five-category birth order/sibling gender variable based on *surviving* siblings. We ran all models with these *living* male sibling categories. While this resulted in some small fluctuations in effect sizes, results were substantively unchanged. Figure 1A presents an illustrative example of the Kaplan Meier curves for breastfeeding duration disaggregated by this variable. Second, our models of breastfeeding initiation omitted children who had died; it is impossible to know whether the children were weaned and subsequently died or died and therefore were no longer breastfeeding. As a robustness check, we re-estimated the models with children who died included. Again, results were substantively unchanged. Finally, because there are a smaller number of children of fourth or higher birth order with no brothers compared to other categories, we coded a version of the variable comparing firstborns, secondborns with an older brother, secondborns without an older brother, third and higher with and older brother, third and higher without an older brother and fitted all models again. There were some minor fluctuations in effect sizes, but results remained substantively unchanged.

## Discussion

There is a large, robust body of evidence documenting negative effects of son preference on girls’ health and survival elsewhere in South Asia [1, 22, 24, 32, 54], but limited literature in Nepal. Here, we investigated: the patterns of mortality by gender across time and place in Nepal; whether girls are disadvantaged in child feeding practices compared to boys; and, where observed, whether gendered feeding practices are associated with mortality risks.

We found geographic variation in mortality differentials: girls face a particular disadvantage in richer, more urban areas. Encouragingly, mortality and nutrition outcomes improved for all children between 1996 and 2016. However, girls were consistently disadvantaged in the post-neonatal period and between ages 1–4 years relative to boys. This is consistent with previous work in Nepal showing boys’ greater biological frailty places them at greater risk of mortality than girls in the neonatal period, but girls face a disadvantage in later periods due to postnatal discrimination [42]. These period-specific patterns explain the seemingly contradictory fact that son preference is high in Nepal, but under-five girls do not face an overall mortality disadvantage.

There are several possible explanations for period-specific effects. First, postnatal discrimination may operate from birth but be insufficient to counterbalance boys’ heightened frailty [55] in the earliest period. Alternatively, parental perceptions/knowledge of infant frailty could encourage strong investments in all children in the first few weeks of life, with a gendered decay over time as infants exit the perceived frail period. This hypothesis is supported by our finding of no gender gap in breastfeeding initiation, which would manifest during this early period, but significant female disadvantage in duration, relevant to later discrimination. Second, parents may be less willing to invest in children perceived as overly frail—it may be that the frailest boys are selected out in the neonatal period due to diminished parental investment. These possibilities are not mutually exclusive. Third, early complementary feeding may be seen as a way of giving favored children (boys) a nutritional advantage. However, such practices would actually place them at greater risk of illness and mortality.

We found no evidence of gender differences in complementary feeding practices, as with elsewhere in South Asia [24–27]. However, we did find a female disadvantage in breastfeeding, with girls with older sisters but with no brothers being most disadvantaged. This finding fits with the theory that son preference manifests not as intentional postnatal discrimination, but rather as asymmetrical preferences for child spacing based on child gender and birth order. Evidence from India [23, 24] and Pakistan [22] shows a very similar pattern by child and sibling gender and birth order, attributed to stopping behavior: Mothers at low parity who want additional children may wean early regardless of child gender, while mothers at higher parity may only wean early to try again if they have not already met gender-specific fertility desires.

Our study has several limitations. First, it is possible boys and girls receive the same *kinds* of food but girls are disadvantaged in the *amount/quality* of food received/are given less nutritious portions, but we could not measure this disparity. For example, if a boy and a girl are usually fed the same curry but the boy systematically receives more pieces of meat and/or leaner, more protein-rich meat while the girl receives less meat and/or fattier, less protein-rich meat, such disparities cannot be measured by the kind of yes/no food category questions available in the NDHS data. Owing to social desirability bias, women may also underreport gender differences. Both factors may introduce a conservative bias in our estimates of postnatal discrimination practices. Second, while extensive literature has measured the impact of son preference, the underlying driver of son preference is arguably the perceived sociocultural and economic value of girls; as with other work in this area, we were unable to directly assess value judgements here. Third, changes in survey design limited our ability to directly compare models across time for some outcomes. Fourth, we were not able to measure exclusive breastfeeding due to data limitations. One cross-sectional study from Bhaktapur (part of the Kathmandu Valley) showed by 1 month of age, one-quarter of infants were no longer exclusively breastfed; by 3 months, only 24% were exclusively breastfed, and by 6 months, the figure dropped to < 10% [56]. It remains unclear whether there are gender disparities in these patterns. To facilitate a critical examination of exclusive breastfeeding practices in future research, nationally representative population data on the duration of exclusive breastfeeding for all children are needed. Fifth, the vast majority of infants are introduced to solid food during a weaning ceremony called pasni [57]. This ceremony occurs earlier for girls, at 5 months, than for boys, at 6 months. We were unable to examine the effect of this cultural practice here as the practice is not directly assessed in the NDHS. In light of continued complementary feeding (in keeping with WHO recommendations), this ceremony would be more relevant to exclusive breastfeeding (not assessed here) than to overall duration, but could also be a factor in gender differences in solid, semi-solid, and soft food consumption. While we did not find evidence of gender differences in food item consumption, future work is needed on whether or how pasni may be associated with gendered nutritional outcomes.

Sixth, many factors beyond nutrition may also help to explain gender differentials in mortality, but we were not able to explore these comprehensively here. Nepal is an extremely varied country, with 126 caste/ethnic groups reported in the 2011 census [43]. Topographically it ranges from the terai (or plains), which has a tropical and subtropical climate to the mountains, which include 8 peaks over 8000 m. As of 2015 Nepal consists of seven provinces, which vary dramatically in terms of their economy, education, and ethnic makeup; for example the Total Fertility Rate in Province 3 is just 1.8, while in Province 2 it is 3.0 [39]. Further work is needed to understand regional and sub-regional differences in the relationships we have identified here. Finally, it should be noted that the government classification of urban and rural areas changed substantially between the 2011 and 2016 rounds of the DHS. According to the 2011 census, 17% of the population lived in urban areas, but the widespread reclassification meant that 59% of the population lived in urban areas by 2016 [39, 43]. It is important to note that this was due to a reclassification of areas rather than actual urbanization. There were many other administrative changes during this time, including expanding the number of municipalities from 58 to 217; as a result it is not possible to recreate the urban/rural classification from 2011 within the 2016 DHS and direct interpretation of this variable should be treated with caution [39, 43].

## Conclusions

The regional patterning of female disadvantage we observed can be used by policymakers to target nutrition interventions to improve girls’ health outcomes in regions where they are most at-risk. Health workers providing counseling during antenatal care (ANC) to improve maternal and infant health [58] provide an opportunity for quality counselling to increase adherence to recommended breastfeeding practices for girls. Such embedded community health workers can identify opportunities for targeted provisioning of early childhood healthcare for girls at greatest risk of undernutrition at mortality. This would complement existing governmental aims to improve the quality of maternal health services and reduce health disparities for women and girls in Nepal [59], as well as being consistent with evidence-based WHO recommendations for expansion of ANC coverage [60]. The Government of Nepal is working to increase universal health coverage and reduce health disparities, but there are considerable inequalities in access to and uptake of quality ANC services [58, 59, 61, 62] and even starker inequalities in postnatal care [63–65].

Our results show girls’ disadvantage is greatest from ages 1–4 years. Community-based health programs, developed in consultation with mothers themselves [60], could be expanded to continue to provide targeted healthcare for children beyond 12 months, with particular focus on nutrition monitoring and health service provision for girls. Nepal’s Female Community Health Volunteers (FCHVs) program could be particularly effective in this aim. Founded in 1988 with a primary focus on family planning but expanded to include maternal and child health services in subsequent decades, the program provides health knowledge and connects local families and communities to health facilities [66]. Although some observers have highlighted gaps in the FCHV program [66], particularly focused on program fragmentation and bureaucratic challenges faced by the volunteers themselves, FCHVs and women’s groups are effective modes of intervention during pregnancy; interventions utilizing these groups have been found to improve maternal and perinatal health [66–69]. Similarly, although some supply-side [70] and demand-side [71] barriers to uptake have been identified in India’s Janani Suraksha Yojana, support services provided by local resident community health workers have been strongly identified as an enabling factor in improving the uptake of maternal health services. The development of strong interpersonal relationships between the community health workers and pregnant women and mothers appear to be a particularly important factor in facilitating better access to and uptake of public healthcare programs in both Nepal and India [69, 71]. The gendered health and nutrition patterns identified here may be reduced through a similar targeting of the nutritional needs of at-risk girls via fostering continuity between ANC and, subsequently, community-based healthcare coverage. Additionally, both to address the need for data on breastfeeding in Nepal, and to inform policy and practice, breastfeeding practices could be recorded by community health workers, anonymized, and made available to researchers to facilitate monitoring of feeding practices and identification of specific at-risk regions and groups.

## Supplementary Information

**Additional file 1.**

## Data Availability

All data are publicly available secondary data available for download from: https://www.dhsprogram.com/Data/.

## Footnotes

1 Lower mean age in 1996 reflects the change in the survey from asking relevant questions for children < 3 years vs. children < 5 years of age noted in the Methods section.
