# Supplementary material for: Gender, nutritional disparities, and child survival in Nepal
Source: BMC Nutr. 2022 May 23;8:50. doi: 10.1186/s40795-022-00543-6 (PMC9125883; doi:10.1186/s40795-022-00543-6)

**Appendix**

Table S1. Multilevel logit models of breastfeeding initiation, all living children, NDHS 1996-2016

|  |  | Model 1 | | Model 2 | | Model 3 | |
| --- | --- | --- | --- | --- | --- | --- | --- |
|  |  | OR | 95% CI | OR | 95% CI | OR | 95% CI |
| Child is Female | | 0.82^**^ | [0.72,0.93] | 0.85^*^ | [0.74,0.98] | 1.00 | [1.00,1.00] |
| Child's Birth Order | |  |  |  |  |  |  |
|  | 1st Born (ref) |  |  |  |  |  |  |
|  | 2nd/3rd Born | 1.80^***^ | [1.51,2.15] |  |  |  |  |
|  | 4th+ Born | 1.48^***^ | [1.19,1.83] |  |  |  |  |
| Birth Order & Sibling Gender | |  |  |  |  |  |  |
|  | 1st Born (ref) |  |  |  |  |  |  |
|  | 2nd/3rd Born, No Brothers |  |  | 1.98^***^ | [1.55,2.54] | 0.95 | [0.74,1.22] |
|  | 2nd/3rd Born, 1+ Brother(s) |  |  | 1.66^***^ | [1.38,1.99] | 2.13^***^ | [1.61,2.83] |
|  | 4th+ Born, No Brothers |  |  | 1.60 | [0.93,2.73] | 1.96^***^ | [1.51,2.54] |
|  | 4th+ Born, 1+ Brother(s) |  |  | 1.17 | [0.90,1.52] | 1.68 | [0.96,2.93] |
| Interactions | |  |  |  |  |  |  |
|  | Female*2nd/3rd Born, No Brothers | |  |  |  | 1.15 | [0.84,1.59] |
|  | Female*2nd/3rd Born, 1+ Brother(s) | |  |  |  | 0.82 | [0.44,1.53] |
|  | Female*4th+ Born, No Brothers | |  |  |  | 0.74 | [0.53,1.04] |
|  | Female*4th+ Born, 1+ Brother(s) |  |  |  |  | 0.81 | [0.24,2.82] |
| Notes: Models control for maternal age and education, urban residence, region, household poverty, survey year, caste/ethnicity, household size, and religion; * p < 0.05, ** p < 0.01, *** p < 0.001 | | | | | | | |

Figure S1. Kaplan Meier Curves for breastfeeding duration for second and higher birth order living children, living older siblings only, NDHS 1996-2006


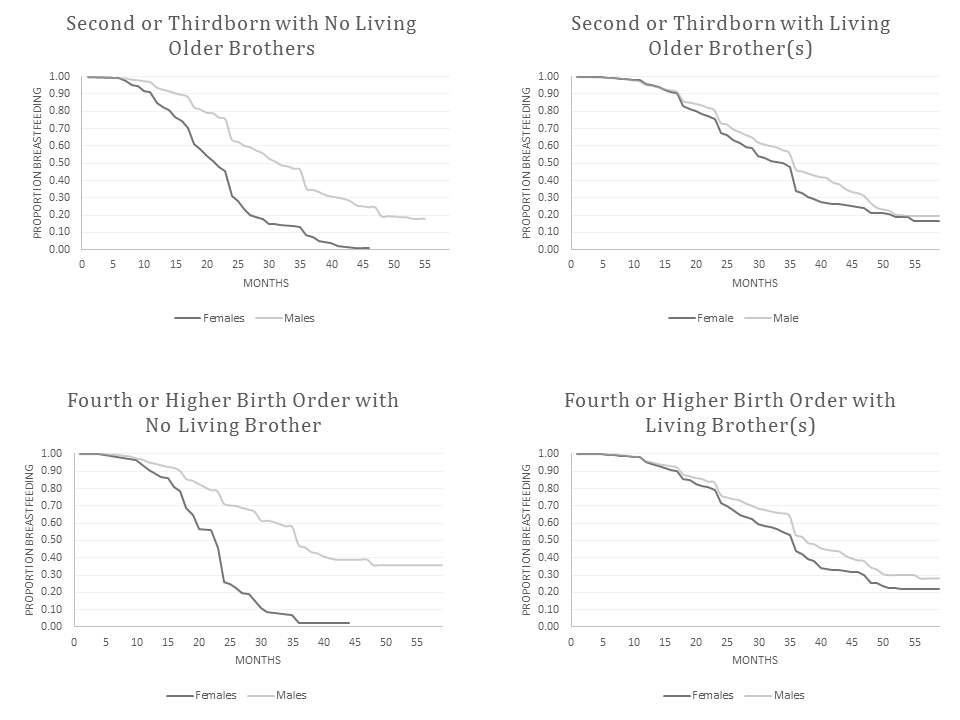

Supplement: Supplementary file 1 — Additional file 1. [file 40795_2022_543_MOESM1_ESM.docx]
